# INTERDEPENDENCE OF CYTOSOLIC PROTEOSTASIS, LYSOSOMAL ACIDIFICATION, AND MITOCHONDRIAL FUNCTION

**DOI:** 10.1093/geroni/igad104.1136

**Published:** 2023-12-21

**Authors:** Chuankai (Kai) Zhou

**Affiliations:** Buck Institute for Research on Aging, Novato, California, United States

## Abstract

Loss of proteostasis, mitochondrial dysfunction, and lysosome defects are conserved hallmarks of aging. One common consequence of cytosolic proteostasis stress is the formation of protein aggregates that are attached to the mitochondrial outer membrane. It remains unknown why cytosolic protein aggregates are attached to the mitochondria. Here we show that Tom70, a conserved receptor for mitochondrial import, moonlights to nucleate the aggregation of cytosolic proteins through some of the newly synthesized mitochondrial proteins. As the cytosolic accumulation of these aggregate-nucleating mitochondrial proteins causes proteostasis stresses, cells balance their import and synthesis through a TOM70-FKH1/2 pathway to regulate the biogenesis of mitochondrial proteins. The reduction of Tom70 during aging causes age-dependent mitochondrial defects in the biogenesis of mitochondrial proteins and attachment of cytosolic protein aggregates, both of which can be rescued by overexpressing TOM70. Additionally, we observed an unexpected rescue of vacuole/lysosome dysfunction in the cells with mitochondrial function preserved during aging. Further investigation revealed a novel conserved mitochondria-to-vacuole/lysosome axis of organelle crosstalk. Our results estabslih novel connections among different cellular compartments related to three hallmarks of aging.

